# Pain and health-related quality of life in patients with hypophosphatasemia with and without ALPL gene mutations

**DOI:** 10.3389/fendo.2022.965476

**Published:** 2022-08-11

**Authors:** Maite Santurtún, Eva Mediavilla-Martinez, Ana I. Vega, Natalia Gallego, Karen E. Heath, Jair A. Tenorio, Pablo Lapunzina, Leyre Riancho-Zarrabeitia, José A. Riancho

**Affiliations:** ^1^ Departamento de Enfermería, Hospital Padre Meni, Universidad de Cantabria, Santander, Spain; ^2^ Facultad de Medicina, Universidad de Cantabria, Santander, Spain; ^3^ Servicio de Genética, Hospital UM Valdecila, Santander, Spain; ^4^ Centro de Investigación Biomédica en Red de Enfermedades Raras (CIBERER), Instituto de Salud Carlos III, Instituto de Genética Médica y Molecular, Hospital U Lapaz, Instituto de Investigación Sanitaria La Paz (IDIPAZ), Madrid, Spain; ^5^ ERN-ITHACA, Brussels, Belgium; ^6^ Servicio de Reumatología, Hospital U Sierrallana Torrelavega, Instituto de Investigación Sanitaria Valdecilla (IDIVAL), Santander, Spain; ^7^ Departamento de Medicina y Psiquiatría, Servicio de Medicina Interna, Hospital Valdecilla, Instituto de Investigación Sanitaria Valdecilla (IDIVAL), Universidad de Cantabria, Santander, Spain

**Keywords:** hypophosphatasia, alkaline phosphatase, quality of life, patient-reported outcomes, pain, disability

## Abstract

**Background:**

Low serum alkaline phosphatase levels are the hallmark of hypophosphatasia, a disorder due to pathogenic variants of the *ALPL* gene. However, some patients do not carry *ALPL* variants and the cause of low alkaline phosphatase remains unknown. We aimed to determine health-related quality of life in adults with low alkaline phosphatase and explore the differences between patients with and without *ALPL* mutations.

**Methods:**

We studied 35 adult patients with persistently low alkaline phosphatase unrelated to secondary acquired causes who had *ALPL* sequenced, and 35 controls of similar age. Three questionnaires about body pain (Brief Pain Inventory, BPI), physical disability (Health Assessment Questionnaire Disability Index, HAQ-DI), and health-related quality of life (36-item Short-Form Health Survey, SF-36) were delivered by telephone interviews.

**Results:**

The mean BPI intensity and interference scores were higher in the patient group (p=0.04 and 0.004, respectively). All domains of the HAQ instrument tended to score better in the control group, with significant differences in the “reach” score (p=0.037) and the overall mean score (0.23 vs 0.09; p=0.029). Patients scored worse than controls in several SF-36 dimensions (Role physical, p=0.039; Bodily pain p=0.046; Role emotional, p=0.025). Patients with and without pathogenic variants scored similarly across all tests, without between-group significant differences.

**Conclusions:**

Patients with persistently low levels of alkaline phosphatase have significantly worse scores in body pain and other health-related quality of life dimensions, without differences between patients with and without pathogenic variants identified in *ALPL* gene. This is consistent with the latter ones carrying mutations in regulatory regions.

## Introduction

Hypophosphatasia is an infrequent disorder due to insufficient alkaline phosphatase activity (1). Four genes encode different isoforms of alkaline phosphatase; three of them encode the tissue-specific forms (placental, intestinal, and germ-cell), and one is the tissue-nonspecific, alkaline phosphatase (*ALPL*), which is expressed in many tissues, including bone, liver, kidney, and teeth. Clinical manifestations of hypophosphatasia are heterogeneous and include musculoskeletal problems (bone, joint, and muscle pain; fractures or pseudo-fractures, tendinopathy, chondrocalcinosis), dental problems (premature loss of teeth), fatigue, abnormal gait, failure to thrive in children, etc. (1–3).

There are several forms of hypophosphatasia, with differences in the age of presentation, severity, and underlying genetic mechanism. In this regard, perinatal and pediatric-onset forms are usually more severe than adult ones. Also, the more severe cases are frequently due to biallelic pathogenic variants and adult-onset Hypophosphatasia is usually heterozygous (4, 5). There is a high degree of allelic heterogeneity, and more than 400 *ALPL* pathogenic variants have been reported so far.

Low serum levels of alkaline phosphatase are the hallmark of hypophosphatasia, which may be accompanied by other biochemical abnormalities, such as mildly increased calcium and phosphate levels, low levels of bone turnover markers, or high levels of alkaline phosphatase substrates, such as pyridoxal-phosphate and phosphoethanolamine. Pyrophosphate is an important substrate for alkaline phosphatase, which hydrolyzes pyrophosphate into phosphate. This is a critical step in bone mineralization. The lack of adequate alkaline phosphatase activity results in the accumulation of pyrophosphate, which inhibits mineralization (1). Unfortunately, pyrophosphate cannot be measured in most centers.

Low serum alkaline phosphatase is a major finding in hypophosphatasia, but it is not pathognomonic. Several acquired conditions (such as acute severe illnesses, malnutrition, magnesium and zinc deficiency, vitamin B12 deficiency, celiac disease, hypercortisolism, or hypothyroidism), drugs (antiresorptives, clofibrate), or a few other hereditary disorders (Wilson disease, cleidocranial dysplasia) can be associated with low alkaline phosphatase levels. Thus, it is imperative to exclude those disorders in the work-up of patients with low alkaline phosphatase (6, 7). If those conditions are excluded, hypophosphatasia is the likely cause of low serum alkaline phosphatase. The diagnosis is confirmed when a pathogenic variant is found in *ALPL*. However, this is not always the case. In several case series, in about one-third to one-half of patients with otherwise unexplained low alkaline phosphatase gene analysis does not reveal a causative genetic defect (8–10).

It is unclear if those patients have hypophosphatasia due to an undetected *ALPL* variant (such as variants in regulatory, intronic region or even epimutations), in other genes not yet associated with HPP, or have a different disorder. Also, it is unclear if there are clinical differences between adult patients with hypophosphatasia (this is, with a deleterious mutation of ALPL) and those with low alkaline phosphatase of unknown cause. Therefore, this study aimed to describe the clinical manifestations (patient-reported outcomes) of a series of adult patients with low alkaline phosphatase, compare them with a control group, and analyze the possible differences between patients with and without *ALPL* pathogenic variants.

## Subjects and methods

### Subjects

Adult patients with persistently low serum alkaline phosphatase were included. They were recruited by a search of laboratory databases as previously reported (8) or among patients sent to our outpatient clinics because of low alkaline phosphatase. Low alkaline phosphatase levels were confirmed in several analysis during periods longer than one year. None of them were taking antiresorptive drugs or other drugs known to impact bone metabolism. An extensive clinical and biochemical study was carried out to exclude endocrine disorders, vitamin D deficiency, malnutrition, celiac disease, anemia or other disorders associated with low alkaline phosphatase. The control group was a convenience sample of volunteer individuals of similar age and sex distribution without severe acute or chronic conditions. The study protocol was approved by the IRB. All participants gave their informed consent to participate in this study. DNA was extracted from patients' blood samples and exons and the flanking intron regions of the *ALPL* gene were analyzed by Sanger sequencing or next-generation sequencing by a custom panel.

### Health-related quality of life instruments

Patient-reported outcomes were evaluated with 3 widely used instruments that have also been previously used to study patients with hypophosphatasia (11). The three questionnaires were filled by telephone interviews of both patients and controls, in Frebruary-April 2022.

The Brief Pain Inventory Short Form (BPI) (12) assesses the severity and impact of pain on daily function (general activity, mood, walking, social relations, sleep, and enjoyment of life). The responses are aggregated into two scores, pain intensity, and pain interference, by computing the mean scores of items related to pain intensity and pain interference, respectively.

The Health Assessment Questionnaire Disability Index (HAQ-DI) is widely used to measure functional status (13). It questions the degree of difficulty in performing 8 activities of daily living (arising, reach, walking, grip, hygiene, dressing, eating, and other activities). A global score is obtained as the mean of the scores of the 8 domains.

The 36-item Short-Form Health Survey Version 2 (SF-36) is a widely used instrument to measure health-related quality of life. The 36 questions cover 8 domains of health: physical functioning (PF, limitations in performing various physical activities); role physical (RP, overall limitations in work and other usual activities); bodily pain (BP, intensity and pain-dependent limitations); general health (GH, respondent views about his/her health); vitality (VT, energy level); social functioning (SF, health-related effects on social activities); role-emotional (RE, mental-health-related role limitations on work and other usual activities; and mental health (MH, anxiety, depression, loss of control and psychological wellbeing). Scores from the 8 domains are aggregated to calculate two component scores: the Physical Component Summary (PCS) and Mental Component Summary (MCS) (14). Patients’ responses were scored using the Quality Metrics Health Outcome Scoring Software (www.qualitymetric.com). The algorithm utilizes norm-based scoring involving a linear T-score transformation so that scores for each domain and component summary have a mean of 50 and a standard deviation of 10, based on the 2009 USA general population. Higher scores indicate better health. Generally, changes <0.3 SD (3 points) are regarded as not relevant.

### Data analysis

After scoring individual responses according to the specific instructions of each questionnaire, the scores of patients and controls were compared using the non-parametric unpaired Mann-Whitney U-test. P-values<0.05 were considered statistically significant. For the comparisons of patients according to their mutational status, we divided them into two groups, one with pathogenic/likely pathogenic variants and the other with benign/likely benign/variants of unknown significance or no identified mutations.

## Results

Among the 51 patients initially identified, 11 could not be contacted after several telephone and postal attempts, 2 declined to participate, and 3 could not complete the interview due to physical or mental problems. Thus, 35 patients and 35 controls were finally studied. Both groups included 8 men and 27 women. The patients’ age was 51 ± 14 yr (range 21-77); the controls’ age was 50 ± 15 yr (range 24-71). In 21 patients (60%) an ALPL variant was identified, which was considered non-pathogenic in 3 cases and pathogenic/likely pathogenic in 18.

**Figure 1:**
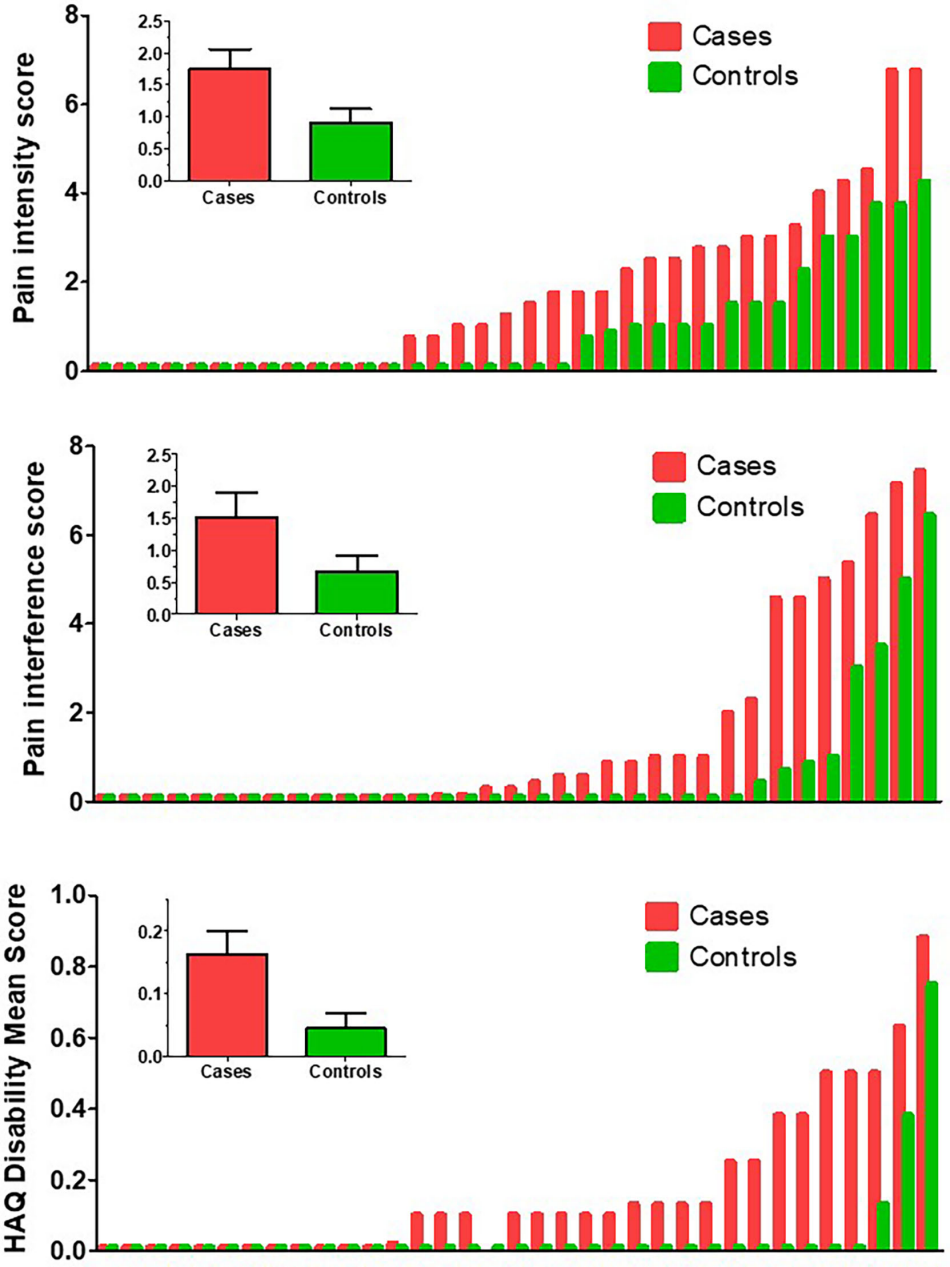
Scores, in ascending order, in patients (red bars) and controls (green bars). Upper panel: Body pain index (BPI) intensity score. Middle panel: BPI interference score. Lower panel: Health Assessment Questionnaire Disability Index (HAQ-DI) mean scores. The insert graph in each panel represents the mean and SEM scores of the cases and control groups.

Body pain, assessed by the BPI, was more intense in the patient group than in the control group. The means of pain intensity score were 1.7 in the patient group and 0.9 in the control group (p=0.042). The mean interference score was 1.5 in the patient group and 0.6 in the control group (p=0.004) (**Figure 1**). Nevertheless, quite a few patients and more controls reported no pain at all (**Figure 1**). As shown in **Table 1**, there were no significant differences in BPI scores between patients with and without ALPL pathogenic mutations.

**Table 1:** Brief pain inventory (BPI) and HAQ scores in patients with and without pathogenic ALPL mutations.

	BPI-Pain intensity		BPI-Pain interference		HAQ mean score	
	No pathogenic variant (n = 17)	Pathogenic variant (n = 18)	No pathogenic variant (n = 17)	Pathogenic variant (n = 18)	No pathogenic variant (n = 17)	Pathogenic variant (n = 18)
Mean	1.9	1.3	1.7	1.3	0.15	0.27
Median	1.7	0	0.9	0	0	0
Minimum	0	0	0	0	0	0
Maximum	4.5	6.7	6.4	7.4	0.5	0.9
p	0.29		0.21		0.31	

The higher the score, the worse pain and disability.

All domains of the HAQ instrument tended to score better in the control group than in the patient group, with significant differences in the “reach” score (p=0.037) and the overall mean score (0.23 vs 0.09; p=0.029) (**Figure 1**). Patients with and without pathogenic mutations showed similar HAQ scores, without significant differences at any individual dimensions or mean scores (p=0.33) (**Table 1**).

**Figure 2:**
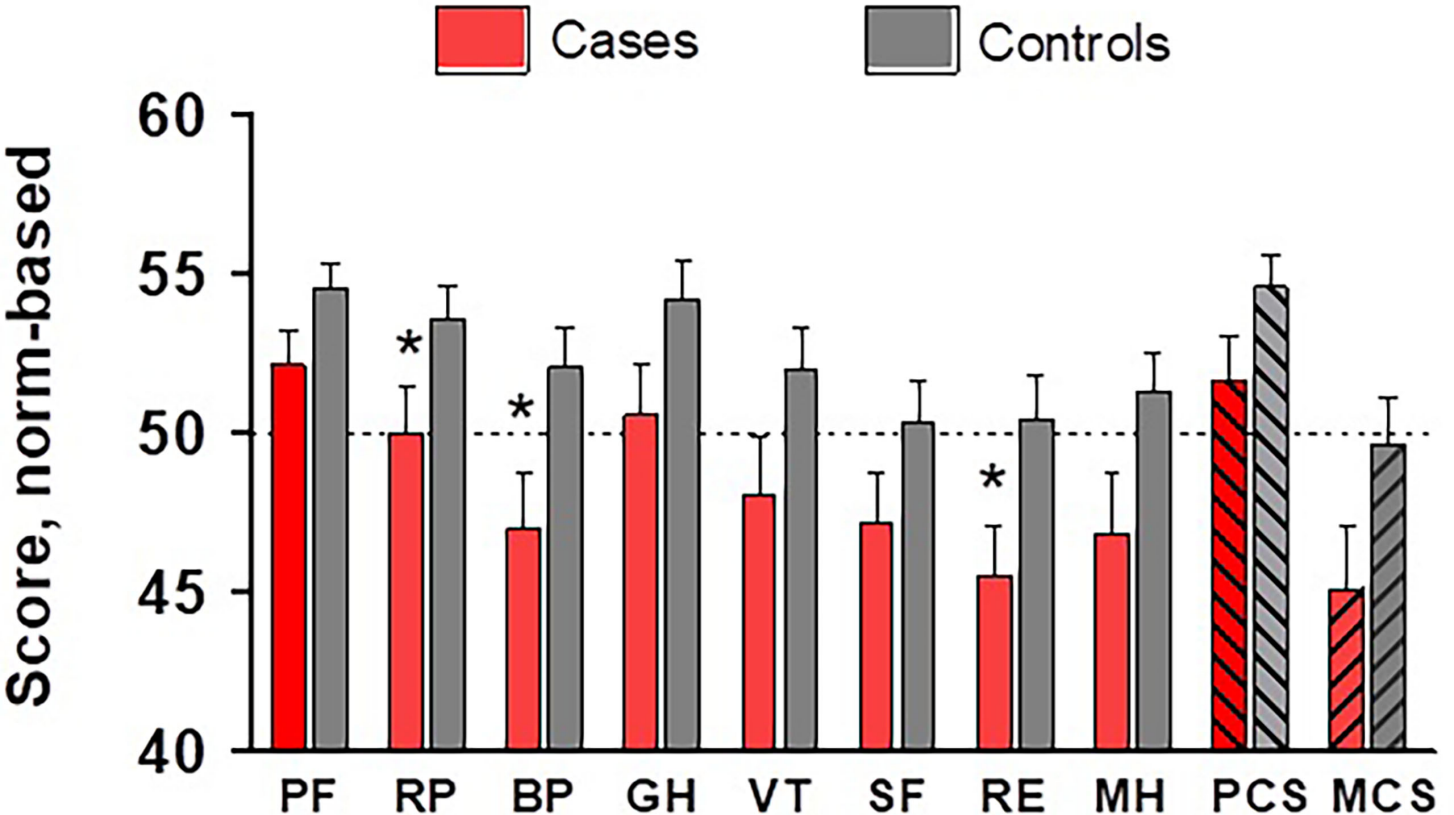
Scores across SF-36 dimensions and global components, normalized against the USA population, in cases (red bars) and controls (grey bars). * p<0.05. PF, physical functioning; RP, role physical; BP, body pain; GH, general health; VT, vitality; SF, social functioning; RE, role emotional; MH, mental health; PCS, physical component summary; MCS, mental component summary.

Regarding the SF-36 tool, several dimensions scored worse in patients than in controls (RP, p=0.039; BP, p=0.046; RE, p=0.025) (**Figure 2**). The proportion of cases and controls with scores<45 in comparison with the reference population (this is, 5 points below the norm) in the RP dimension was 23% and 11%, respectively; 43% and 17% in BP; and 40% and 26% in RE. Patients with and without mutations scored similarly across all dimensions, without between-group significant differences (**Table 2**).

**Table 2:** SF-36 scores (norm-based) in cases with and without ALPL mutations.

	RP (Role physical)		BP (Body pain)		RE (Role emotional)		
	No pathogenic variant (n = 17)	Pathogenic variant (n = 18)	No pathogenic variant (n = 17)	Pathogenic variant (n = 18)	No pathogenic variant (n = 17)		Pathogenic variant (n = 18)
Mean	49.1	50.8	43.7	50.8	46.1		44.9
Median	52.7	54.9	50.1	50.7	45.7		45.7
Minimum	30.2	25.7	30.1	26.5	35.3		21.3
Maximum	57.2	57.2	62.0	62.0	56.2		56.2
p	0.37		0.06			0.86	

For simplicity, only dimensions showing significant differences between cases and controls are shown. The lower the score, the worse quality of life.

## Discussion

The clinical spectrum of hypophosphatasia is wide and clinical manifestations go from very severe, even lethal, perinatal cases to cases with only mild symptoms. Specifically, adult-onset cases tend to be milder than pediatric-onset cases. Hypophosphatasia in adults frequently presents with dental problems, stress fractures, tendinopathy, or other musculoskeletal complaints (2, 5). Thus, the burden of disease in pediatric cases is well-recognized, but health complaints in adult cases have not been well established.

In this study, we used three well-known tools to explore the health-related quality of life in patients with persistently low alkaline phosphatase, the hallmark of hypophosphatasia (11, 15–17). Even though the group was comprised of individuals without evidence of severe skeletal problems, they scored worse than age-matched controls in several items related to body pain, in all the three questionnaires used (BPI, HAQ, and SF-36). Although the intensity of the manifestations appeared to be mild, and significantly less severe than those in the pediatric-onset series (11, 15), the results were consistent across the three questionnaires. Likewise, some emotional issues were apparent from the SF-36 data, perhaps reflecting some subjective ailments and discomfort related to subtle disease manifestations.

Nutritional deficiencies and other acquired disorders should be excluded in patients with low alkaline phosphatase. Then, the diagnosis of hypophosphatasia can be confirmed by finding a pathogenic variant in *ALPL*. However, that only happens in about one-third to one-half of patients with low alkaline phosphatase without other obvious causes (8–10). The reason for the low enzyme activity in those patients is unclear. Perhaps they have allelic variants in other genes modulating alkaline phosphatase expression. Alternatively, they may carry genetic variants in deep intron regions or in regulatory regions of the ALPL gene (that are not usually explored when the exonic regions are sequenced or in custom panels), or the accumulation of common functional polymorphisms (18).

Interestingly, in this study, we did not find differences in health-related quality of life scores in patients with or without pathogenic variants. This is also in line with previous reports showing similar levels of bone turnover markers and other biochemical parameters in patients with low alkaline phosphatase with or without ALPL pathogenic variants (10, 19). Overall, both patient subgroups seem to be homogeneous and part of a similar disease spectrum, which would be consistent with the hypothesis that most patients with hypophosphatasemia of unknown cause may have an unidentified variant in *ALPL*. However, since DNA methylation is an important factor regulating ALPL expression (20), epimutations cannot be excluded as a potential explanation for the low alkaline phosphatase levels.

This study has some limitations. First, given the remote nature of the interviews, we do not have objective measures of physical function, such as muscle strength or walking speed. Second, the questionnaires were delivered by telephone, which may have somewhat influenced the responses. Some studies showed that the telephone mode of administration of SF-36 involves an interviewer effect that tends to improve the scores, in comparison with the self-completion method (21). However, other studies found that both methods are equivalent and valid (22). In any case, the same method was used with patients and controls. Third, we did not select a random sample of population controls, which may potentially introduce some selection bias. However, our controls scored similarly to other reference populations in HAQ (13). Also, the average scores of the patient group were lower than those of the USA reference population in several dimensions of the SF-36 questionnaire, with differences over 3-5 points which are usually considered relevant in group comparisons (23, 24). It is to note that even 1-point lower scores in some SF-36 scales have been associated with an excess risk of up to 9% for mortality and 12% for inability to work in some studies (25).

In conclusion, this study shows that patients with persistently low levels of alkaline phosphatase have significantly worse scores in body pain and other health-related quality-of-life dimensions. However, we found no differences between carriers and non-carriers of pathogenic ALPL variants. In theory, this would be consistent with the latter ones carrying mutations in regulatory, not sequenced, gene regions.

## Data availability statement

The raw data supporting the conclusions of this article will be made available by the authors, without undue reservation.

## Ethics statement

The studies involving human participants were reviewed and approved by Comite de ética de investigación con medicamentos de Cantabria. The patients/participants provided their written informed consent to participate in this study.

## Author contributions

Study conception and coordination: JR and LR-Z. Data acquisition: MS, EM-M, JT, KH, NG, AV, and PL. Data analysis: JR, JT, and EM-M. Manuscript scientific input: All. All authors contributed to the article and approved the submitted version.

## Funding

The genetic analysis was funded in part by a research grant from Alexion.

## Conflict of interest

The authors declare that the research was conducted in the absence of any commercial or financial relationships that could be construed as a potential conflict of interest.

## Publisher’s note

All claims expressed in this article are solely those of the authors and do not necessarily represent those of their affiliated organizations, or those of the publisher, the editors and the reviewers. Any product that may be evaluated in this article, or claim that may be made by its manufacturer, is not guaranteed or endorsed by the publisher.
